# Violin Bridge Mobility Analysis under In-Plane Excitation

**DOI:** 10.3390/s131115290

**Published:** 2013-11-08

**Authors:** Cheng-Zhong Zhang, Guang-Ming Zhang, Bang-Yan Ye, Li-Dong Liang

**Affiliations:** 1 School of Mechanical and Automotive Engineering, South China University of Technology, No. 381, Wushan Road, Tianhe District, Guangzhou 510640, China; E-Mails: zcz_505@163.com (C.-Z.Z.); byye@scut.edu.cn (B.-Y.Y.); grups1988@163.com (L.-D.L.); 2 School of Software Engineering, South China Normal University, Nanhai Campus, Foshan, Guangdong 528225, China; 3 General Engineering Research Institute, Liverpool John Moores University, Byrom Street, Liverpool L3 3AF, UK

**Keywords:** violin bridge, frequency response, contact stiffness, dynamic contact vibration, dynamometer

## Abstract

The vibration of a violin bridge is a dynamic contact vibration with two interfaces: strings-bridge, and bridge feet-top plate. In this paper, the mobility of an isolated bridge under in-plane excitation is explored using finite element modeling based on the contact vibration model. Numerical results show that the dynamic contact stiffness in the two contact interfaces has a great impact on the bridge mobility. A main resonance peak is observed in the frequency range of 2–3 kHz in the frequency response of the isolated bridge when the contact stiffness is smaller than a critical threshold. The main resonance peak frequency is affected by the contact stiffness as well. In order to verify the numerical findings, a novel experimental system is then designed on the basis of a piezoelectric dynamometer for bridge mobility analysis. Experimental results confirm the impact of the dynamic contact stiffness on the bridge mobility.

## 1. Introduction

Measuring and studying the violin bridge mobility is not a new topic. Numerous experimental studies of bridge motion have been carried out in the past. For example, in [1], the bridge motility was measured when a bridge is in isolation and in a violin. The experimental results showed that the string resonances excite the violin body resonances at low frequencies, and the main bridge resonance at higher frequencies. At the resonance frequency the admittance of the bridge may become approximately as large as the specific string admittance, implying that complex phenomena such as coupling between the bridge and the string resonances may occur. In [2], the experimental results based on the plate solid bridges show that the distance between the bridge feet has a profound effect on the overall response of a violin. A diminished distance will lower the bridge hill frequency, but increase the bridge hill level. In [3], the effect of the chemical treatments of the bridge on the vibration mode frequency and damping changes was studied. Some theoretical models have also been developed to predict the bridge mobility in the literature [4,5]. In [4], a theoretical model has been developed to investigate the bridge hill. Parameter studies were presented which reveal how the bridge hill is affected by the properties of the bridge and violin body. The model prediction is in fairly good agreement with the experimental findings published in the literature. In [6], a comprehensive study of the violin bridge filter role has been carried out through modal and acoustic measurements. A brief review on the major findings of previous research work can be found in [6]. Finite element analysis was used for modal analysis of violin bridges for investigating the effect of wood removal on the bridge in-plane and out-of-plane vibrating modes [7]. In the literature, the dynamic response of a violin is often divided into a deterministic region and a statistic region [5,8]. The deterministic region is a range of low frequencies in which easily separable natural frequencies can be examined deterministically, and the statistic region is the higher frequencies at which only statistical statements are possible in the frequency domain. In the statistic region, modes overlap significantly, and mode tracking becomes problematical. In this paper the mobility of an isolated bridge is studied numerically and experimentally on the basis of the dynamic contact vibration model. The impact of the contact stiffness on the bridge mobility is investigated.

## 2. Dynamic Contact Vibration Model of the Violin Bridge

The vibration of the bridge when it is mounted in a violin is a dynamic contact vibration with two interfaces: strings-bridge, and bridge feet-top plate. According to the Hertzian contact vibration theory, the contact stiffness changing can cause the bridge resonance frequency shift and the resonance amplitude changing for each vibration mode. The influence of the dynamic contact stiffness on the bridge mobility has not been studied comprehensively up to now.

The dynamic contact vibration of a rigid punch on an elastic medium has been studied by many researchers [9–12]. Most researchers considered the contact to be equivalent to an elastic-spring support and adopted the Hertzian static-contact stiffness. Different dynamic Hertzian contact models based on a nonlinear mass-spring-damping system have been presented to investigate its nonlinear vibration theoretically and experimentally. In [11], an analytical model based on a linear-elastic theory for dynamic contact stiffness of a vibrating rigid sphere contacting a semi-infinite viscoelastic solid was proposed. The dynamic contact-pressure distribution at the interface between the rigid sphere and the viscoelastic solid was deduced first. Then, the dynamic contact stiffness at the interface was deduced from the approximate dynamic contact boundary conditions for displacements. In [12], experimental results showed that the contact stiffness not only affects the resonance frequency position, but also the amplitude of the resonance.

When a bridge is fitted in a violin, the contact stiffness in the two contact interfaces of strings-bridge and bridge feet-top plate is affected by a variety of factors such as the force generated by the strings, the materials and the surface roughness of both the bridge and the top plate, and the area of the contact surfaces. Moreover, the violin top plate dynamic vibration and the string dynamic vibration will change the contact stiffness dynamically. Thus, the contact stiffness is affected by the interaction between the string, bridge and top plate. In addition, some of the plate structure changes may be very sensitive to the bridge-corpus interaction, for example the cutting of the slot-like f-holes which are close to the bridge feet. Thus, the dynamic contact stiffness is sensitive to such structure changes.

Evidently, in this paper the term isolated bridge does not mean the bridge is completely independent of the strings and the corpus. In fact, the bridge is linked to the strings and corpus through the dynamic contact stiffness. However, the dynamic contact stiffness is difficult to determine analytically. Moreover, the violin bridge vibration is very complicated with many vibration modes. An accurate analytical dynamic contact vibration model is difficult to deduce to predict the bridge mobility. The impact of the dynamic contact stiffness on the bridge mobility is studied through finite element modeling and experimental measurements in this paper.

## 3. Finite Element Modeling of a Violin Bridge under the Contact Vibration Model

**Figure 1.**
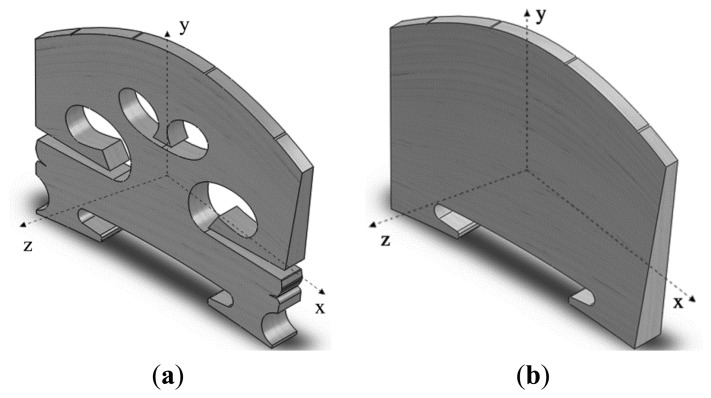
Geometrical models. (**a**) A real bridge; (**b**) A plate solid bridge.

Frequency response analysis of a violin bridge is carried out using ANSYS Workbench 12. The bridge geometric model in Figure 1a was built using the CAD software SOLIDWORKS according to the physical parameters of a real violin bridge. The maximum length, height and thickness of the bridge are 49.5, 34.5 and 4.5 mm, respectively. The top edge width of the bridge is 2 mm. The bridge material is maple. The material properties of ‘maple red’ published in [13] were used in the simulation as listed in Table 1, where the X, Y, Z directions are as defined in Figure 1. No pre-stress has been considered in any of the simulations of this paper.

**Figure 2.**
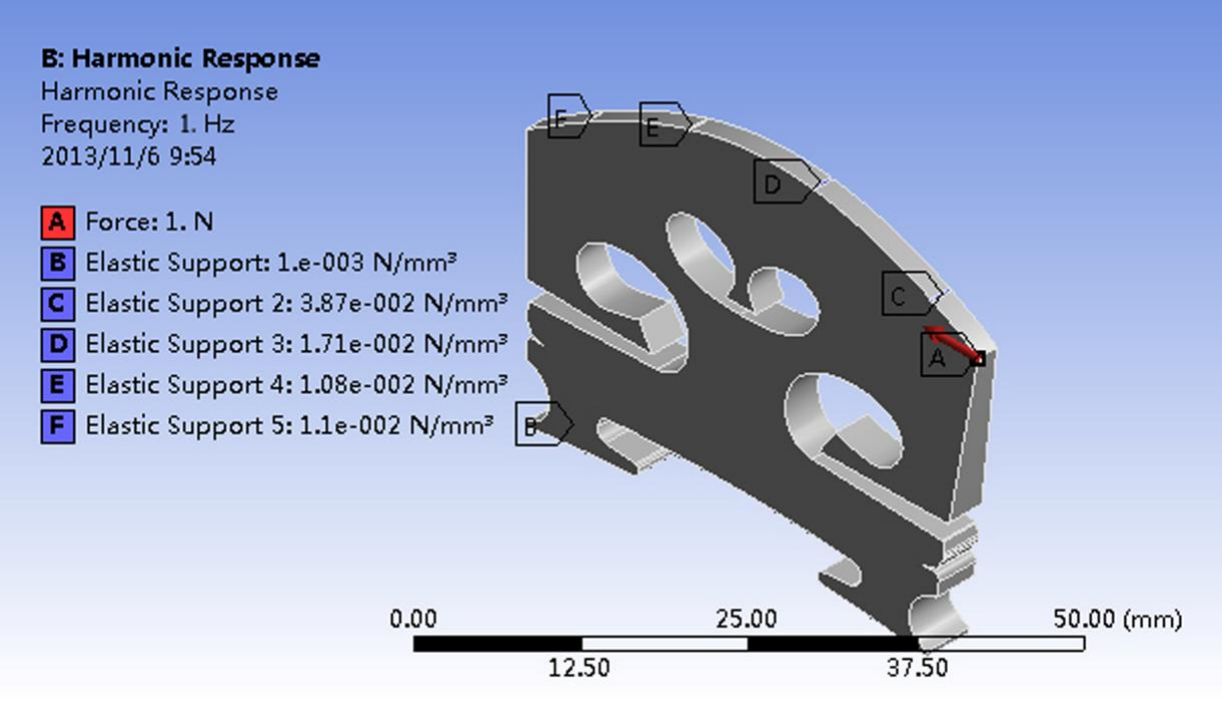
The configuration of the elastic supports and driving force used for numerical analysis of the bridge contact vibration.

Elastic supports (elastic support B in Figure 2) were applied to the bottom surfaces of the two bridge feet, which are the contact surfaces between the two bridge feet and violin top plate, and the elastic foundation contact stiffness in this contact interface is denoted as EFS1. Elastic supports were also applied to the groove/notch surfaces of the bridge top, which are the contact surfaces between the bridge and four strings. In a violin the strings are just placed on the arched notches of the bridge top. Thus, the elastic supports in the notch surfaces are appropriate to model the constraints exerted by the strings. According to the contact mechanics theory [14,15], which studies the deformation of solids that touch each other at one or more points, the contact vibration involves compressive and adhesive forces in the direction perpendicular to the interface, and frictional forces in the tangential direction. These interface forces act on the bridge notch surfaces, constraining the bridge dynamic motion.

The elastic foundation contact stiffness in the contact interface between the strings-bridge top is denoted as EFS2. The elastic supports at the G, D, A, and E strings are indicated as elastic support C, D, E, and F, respectively, in Figure 2. This configuration is equivalent to the violin bridge sitting on a violin with four strings fitted. However, the string and the corpus resonances have been isolated to the frequency response analysis of the isolated bridge, facilitating the study of the bridge behaviour. No constraints were applied to any other surfaces of the bridge, such as the side of the bridge feet.

A sine driving force of 1N was applied to the bridge G-corner in its plane in the bass-bar side as shown in Figure 2 (red arrow A). The Y-directional acceleration response was measured on the bridge foot (using average) in the sound-post side in all the simulation results in this paper except in Section 4.1. The damping ratio was set as 0.7% of critical according to the experimental measurements published in [3].

EFS is defined in ANSYS as spring stiffness per unit area that only acts in the direction normal to the face of the element. When the surface is planar and the loading acts normal to the direction, EFS is defined as:

(1) $$\text{EFS}=\text{F}/(\text{Area}\cdot {\text{Y}}_{\text{disp}})$$

where ‘Area’ is the area of the contact surface, and Y_disp_ is the displacement at the location of EFS due to the loading force F. From Equation (1), it can be seen that the dynamic contact stiffness is affected by a variety of factors, as discussed in Section 2.

## 4. Bridge Mobility Analysis

### 4.1. Mobility Analysis of an Isolated Bridge Based on the Fixed Support Model

**Figure 3.**
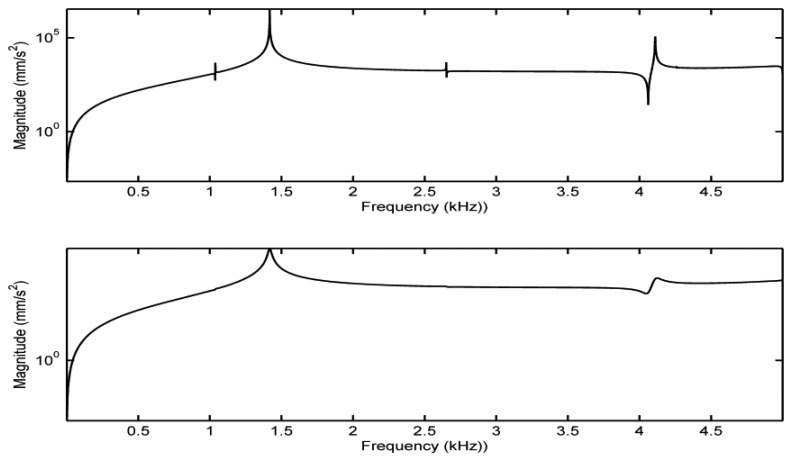
Frequency responses of an isolated bridge with the bridge feet clamped. Top: damping ratio 0.017% critical; Bottom: damping ratio 0.7% critical. The two peak frequencies in the bottom plot are 1,417 Hz and 4,084 Hz, respectively.

For comparison purposes, before we explore the bridge mobility under the contact vibration model, we first study the bridge mobility based on the fixed support model. In this case, fixed supports are applied to the bottom surface of the two bridge feet. No other constraints are applied to the bridge. This configuration is equivalent to the isolated bridge being clamped at the two bridge feet, which is a configuration often used to measure the mobility of an isolated bridge experimentally in the literature [6]. The driving force is the same as described in Section 3. Notice that with the fixed supports, acceleration responses cannot be measured from the bridge feet. The Y-directional acceleration responses measured on the bridge G-corner in the sound-post side are shown in Figure 3 with the damping ratio set as 0.017% critical and 0.7% critical, respectively. From Figure 3, it can be seen if we use the damping ratio of 0.7% critical measured experimentally in [3], the minor resonances disappear but the overall shape is the same. For the fixed support model, no peaks in the bridge mobility are observed in the frequency range of 1.5–4 kHz when the damping ratio is 0.7% critical.

**Figure 4.**
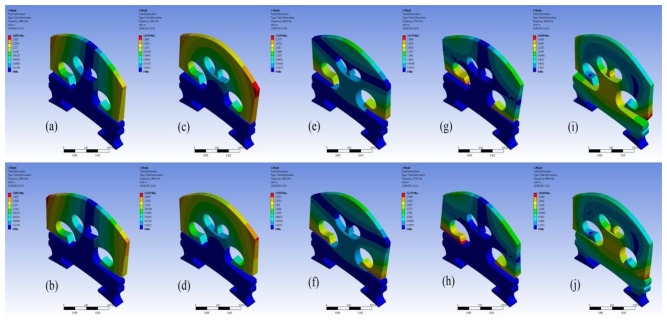
Bridge motion of different resonance modes in Table 2. (**a**) and (**b**): mode 2; (**c**) and (**d**): mode 3; (**e**) and (**f**): mode 4; (**g**) and (**h**): mode 5; (**i**) and (**j**): mode 7. Note that we attach no significance to the amplitude of the motion.

In order to understand the bridge mobility, modal analysis was carried out under the same configuration. The first ten-order natural resonant frequencies of the bridge with the bridge feet clamped are listed in Table 2. In Table 2, ‘O’ indicates the mode is out-of-plane vibration, and ‘I’ is an in-plane vibration. It was found that most of the resonance modes are out-of-plane vibration modes although the driving force in the simulation is in-plane. This is due to the fact that the violin bridge is not truly symmetrical. We then examined the motion of the resonance modes in Table 2. Figure 4 shows the motion of six vibration modes. Each vibration mode is visualized by two images extracted from the animation in ANSYS modal analysis in order to intuitively show the bridge movement of that mode. The two images for each mode show the bridge motion with the maximum displacement in two opposite positions, respectively. From Figure 4 and Table 2, it can be seen that the main resonance peak at 1.4 kHz in Figure 3 is mainly attributed to the in-plane rocking mode 3.

### 4.2. Bridge Mobility Analysis Based on the Contact Vibration Model

**Figure 5.**
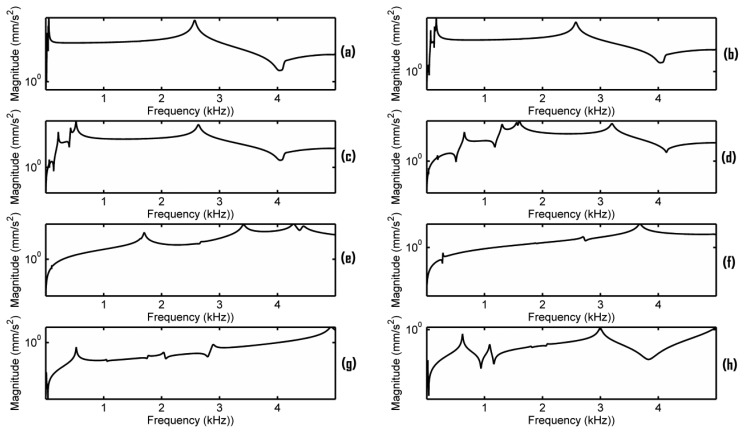
Frequency responses of the bridge in Figure 1a under different contact stiffness with the damping ratio 0.7% critical. EPS1 was set as (**a**) 0.001; (**b**) 0.01; (**c**) 0.1; (**d**) 1; (**e**) 10; (**f**) 100; (**g**) 1,000; (**h**) 5,000 N/mm^3^. The peak frequencies in the frequency range of 2–3 kHz in Figure 5a–c are 2,570, 2,576, and 2,630 Hz, respectively.

In this paper we do not intend to simulate a real dynamic contact vibration process of the bridge. The dynamic contact vibration process is studied by changing the elastic foundation contact stiffness. Each frequency response is obtained based on the contact vibration model. Figure 5 shows the acceleration responses *versus* different elastic foundation contact stiffness using the simulation configuration described in Section 3. EFS1 is changed from 0.001 to 5,000 N/mm^3^. We denote the elastic foundation contact stiffness at the G, D, A, and E string positions as EFS2_G, EFS2_D, EFS2_A, and EFS2_E, respectively. In a real violin, EFS1 and EFS2 are correlated to each other according to Equation (1). The area for all the contact surfaces can be assumed to be approximately constant and the force in Equation (1) is the main factor that affects EFS1 and EFS2 dynamically. The sum of the forces exerted on the strings–bridge contact interface by the strings will pass to the bridge feet-top plate contact interface. Thus, in the simulation of Figure 5, EFS2 was set on the basis of EFS1 as, EFS2_G = C_G_·EFS1, EFS2_D = C_D_·EFS1, EFS2_A = C_A_·EFS1, and EFS2_E = C_E_·EFS1. Suppose the static forces generated by the G, D, A, and E strings are 22.05, 20.68, 21.85 and 26.75 N, and their diameters are 0.75, 0.72, 0.48, 0.26 mm respectively. The contact area in Equation (1) is calculated using the geometrical model in Figure 1a. Assume Y_disp_ to be the same at all the contact surfaces. The ratios C_G_ = 11, C_D_ = 10.8, C_A_ = 17.1 and C_E_ = 38.7 were then estimated using Equation (1). In the estimation, the dynamic force (about 1 N) was ignored because it is much smaller than the static forces.

From Figure 5, it can be seen that the elastic foundation contact stiffness has a significant impact on the bridge mobility. This can be explained by the contact vibration model and modal model described in [16]. As described in Section 2, the contact stiffness not only affects the resonance frequency position but also the amplitude of the resonance. Moreover, the bridge mobility is the superposition of the modal amplitudes.

**Figure 6.**
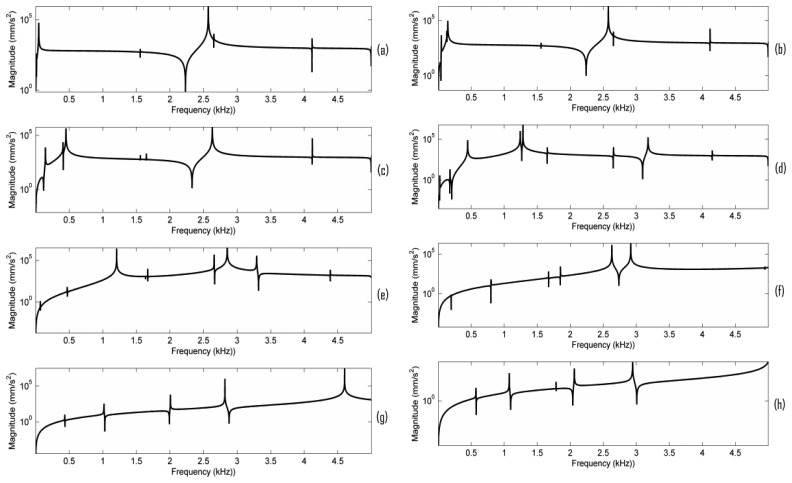
Frequency responses of the bridge in Figure 1a under different contact stiffness with the damping ratio 0.017% critical. EPS1 was set as (**a**) 0.001; (**b**) 0.01; (**c**) 0.1; (**d**) 1; (**e**) 10; (**f**) 100; (**g**) 1,000; (**h**) 5,000 N/mm^3^.

In order to observe the minor resonances in the bridge mobility, the acceleration responses when the damping ratio is set as 0.017% critical are plotted in Figure 6. From Figures 5 and 6, it can be seen that when EFS1 is smaller than a threshold (here 0.1 N/mm^3^), the frequency response is fairly stable, except in the very low frequency range, and a main resonance peak in the frequency range of 2–3 kHz is observed. Our simulation results also indicate that if EFS1 is small enough (here 0.001 N/mm^3^), the main peak frequency becomes almost constant (2,570 Hz here). Between 0.001 to 0.1 N/mm^3^, the main peak frequency changes slightly. When EFS is between 0.1 to 1,000 N/mm^3^, the bridge mobility is very unstable. When EFS1 is bigger than 5,000 N/mm^3^, the bridge mobility seems tending to be stable as well in Figure 6, although without a main resonance peak in the 2–3 kHz frequency range.

Notice that during violin playing, the contact stiffness will change dynamically. Assume the EFS1 will change from 0.001 to 0.1 N/mm^3^. The dynamic response of the bridge will be a mixture of the frequency responses obtained by EFS1 changing from 0.001 to 0.1 N/mm^3^. Since the main resonance peak frequency in the frequency range of 2–3 kHz changes slightly in these instant frequency responses, as seen in Figures 5a–c, the dynamic response of the bridge will most likely exhibit as a broader resonance peak than as the main resonance peak observed in any instant frequency response in Figure 5a–c. Furthermore, if all the dynamic EFS1 values are small enough (smaller than 0.1 N/mm^3^ here), the main resonance peak is more predictable. However, if the dynamic EFS1 ranges from 0.1 to 1,000 N/mm^3^, the dynamic response of the bridge will be very difficult to predict because the instant frequency responses are very unstable, as seen in Figures 5 and 6. As a result, the broad peak in the dynamic response of the bridge is difficult to predict deterministically because of the dynamic contact stiffness. It might be interesting to explore in the future if there is a link between the phenomena observed here and the statistic region in the violin dynamic response.

In order to further understand the bridge mobility, modal analysis was carried out under the same configuration as in Figure 5. The vibration modes close to 2.6 kHz are listed in Table 3. When EFS1 is smaller than 0.1 N/mm^3^ or larger than 1,000 N/mm^3^, the vibration modes are quite stable, except at very low frequency. From Figure 6 and Table 3, it can also be seen that the bridge mobility is linked to the bridge vibration modes. However, the main resonance peak frequency, for example 2,570 Hz in Figure 5a, is not exactly equal to the natural resonance mode frequency of 2,616 Hz since the main resonance peak in the bridge mobility is affected by the neighbouring natural resonance modes as well.

**Figure 7.**
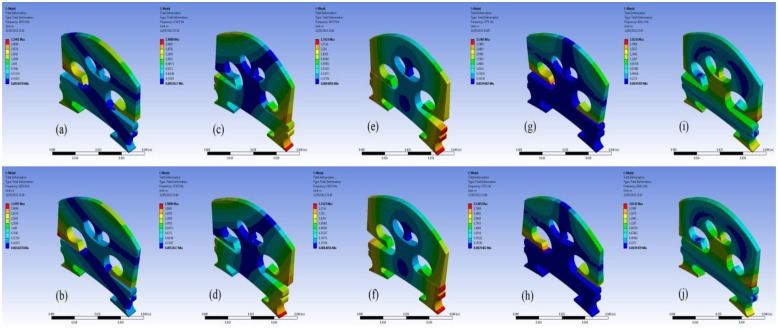
Bridge motion of the five major resonance modes at 1,654 Hz (**a**,**b**), 1,720 Hz (**c**,**d**), 2,616 Hz (**e**,**f**), 2,775 Hz (**g**,**h**), and 4,241 Hz (**i**,**j**) in the case of EFS1 = 0.001 N/mm^3^ in Table 3. Note that we attach no significance to the amplitude of the motion.

We further examined the motion of the resonance modes for the case of EFS1 = 0.001 N/mm^3^ in Table 3. It can be observed that most of the resonance modes are out-of-plane vibration modes. Figure 7a–j shows the motion of the five vibration modes at 1,654 Hz, 1,720 Hz, 2,616 Hz, 2,775 Hz, and 4,241 Hz for the case of EFS1 = 0.001 N/mm^3^ in Table 3. Like in Figure 4, each vibration mode is intuitively visualized by two images extracted from its vibration animation in the ANSYS modal analysis. From Figure 6a, we can see that the five vibration modes have a major contribution to the bridge mobility. Figure 7e,f shows the motion of the resonance mode at 2,616 Hz, which is an in-plane rocking vibration. From Figure 6a, it can be seen that the main resonance peak in the frequency range of 2–3 kHz is mainly attributed to this in-plane rocking vibration. From the rocking motion, it is observed that the upper part and lower part of the bridge behave like two seesaws rocking in opposite directions with the bridge waist as the central axis. Due to the bridge cut-outs, the thin waist makes this rocking mode easier to be excited by the string vibration. Moreover, it can be observed that all five vibration modes in Figure 7 are linked to the cut-outs. Therefore the cut-outs in a bridge are important. In addition, it can be seen that for the same motion in Figures 4 and 7, its natural resonance frequency may be very different. For example, the natural resonance frequency of in-plane rocking motion in Figure 4 is 1,441 Hz, but it is 2,616 Hz in Figure 7. However, the natural resonance frequency of the mode 7 in Figure 4 is 4,384 Hz, while for the same bridge motion in Figure 7 it is 4,241 Hz, and the difference is much smaller. This means that some vibration motions are heavily affected by the dynamic contact stiffness. Thus, the dynamic contact vibration model is more suitable to study the bridge mobility than the fixed support model.

**Figure 8.**
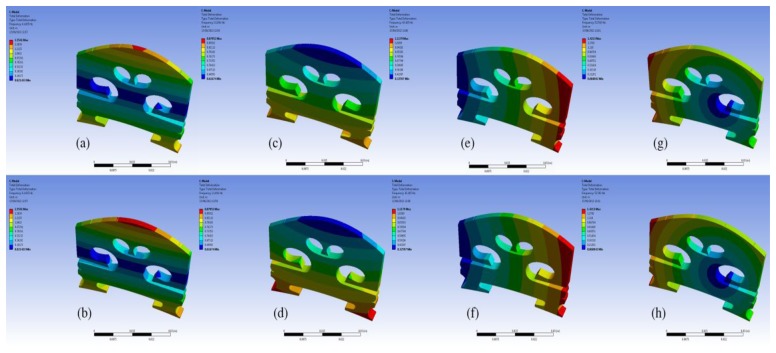
Bridge motion of the four lower frequency resonance modes at 6 Hz (**a**,**b**), 14 Hz (**c**,**d**), 41 Hz (**e**,**f**), and 46 Hz (**g**,**h**) in the case of EFS1 = 0.001 N/mm^3^ in Table 3. Note that we attach no significance to the amplitude of the motion.

From Figures 5 and 6, it is also observed that the frequency responses in the very low frequency region are very unstable. All the resonance modes in this region (for example, the resonance modes lower than these modes shown in Figure 7 for the case of EFS1 = 0.001 N/mm^3^ in Table 3) are rocking as a rigid body as shown in Figure 8. The results in Figure 7 and Figure 8 are consistent with the concept that ‘in the deterministic region the bridge rocked approximately as a rigid body, while in the statistical region bridge motions can be characterized as quiet-feet/rocking-wiggling top’ [6]. The impact of these low frequency resonance peaks on the violin acoustic performance needs further investigation in the future.

**Figure 9.**
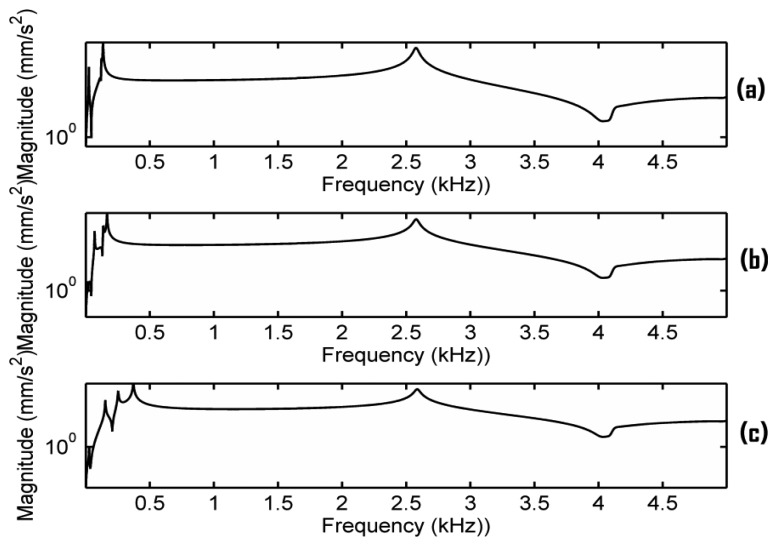
Frequency responses of the bridge in Figure 1a under different contact stiffness with the damping ratio 0.7% critical when EPS1 = 0.01 and (**a**) C_G_ = 1.1, C_D_ = 1.08, C_A_ = 1.71, C_E_ = 3.87; (**b**) C_G_ = 11, C_D_ = 10.8, C_A_ = 17.1 and C_E_ = 38.7; (**c**) C_G_ = 110, C_D_ = 108, C_A_ = 171 and C_E_ = 387.

Finally, additional simulations were carried out to examine the effect of the inaccuracy of the estimated ratios C_G_ = 11, C_D_ = 10.8, C_A_ = 17.1 and C_E_ = 38.7. We redid all the simulations of Figure 5 using the averaged ratio to replace the estimated ratios, *i.e.*, C_G_ = C_D_ = C_A_ = C_E_ = 19.4, and almost the same results were obtained. It seems that the frequency responses are not very sensitive to these ratios. Further simulations were carried out to verify this finding. Figure 9 presents the results when keeping EFS1 = 0.01 N/mm^3^ and changing the ratios C_G_, C_D_, C_A_, C_E_ to set EFS2. Figure 9a shows the result of C_G_ = 1.1, C_D_ = 1.08, C_A_ = 1.71 and C_E_ = 3.87. Figure 9b shows the result of using the estimated ratios. Figure 9c shows the result of C_G_ = 110, C_D_ = 108, C_A_ = 171 and C_E_ = 387. The additional simulations confirms that the main resonance peak in the frequency range of 2–3 kHz is not very sensitive to these ratios when EFS1 < 0.1. In other words, the main resonance peak can be generated in relative flexible conditions for the bridge in Figure 1a.

### 4.3. Mobility Analysis of a Plate Solid Bridge Based on the Contact Vibration Model

**Figure 10.**
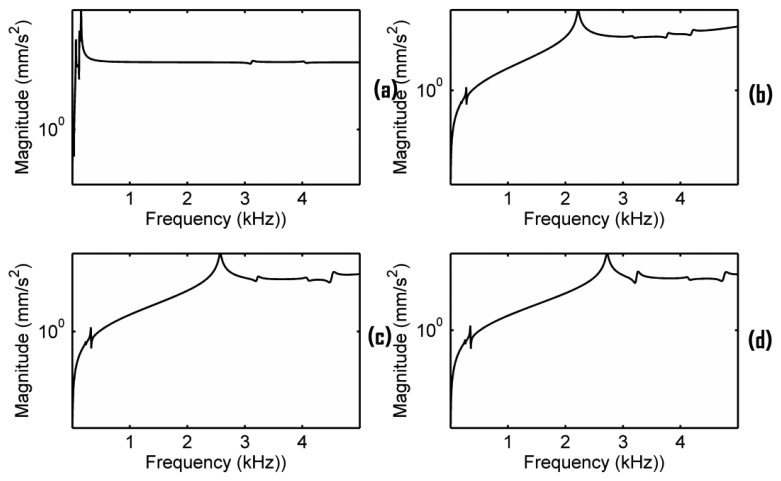
Frequency responses of the plate solid bridge in Figure 1b with the damping ratio 0.7% critical when EFS1 = 0.01 N/mm^3^ and (**a**) EFS2 using the estimate ratios; (**b**) EFS2 = 1,000 N/mm^3^; (**c**) EFS2 = 1,600 N/mm^3^; (**d**) EFS2 = 2,000 N/mm^3^.

**Figure 11.**
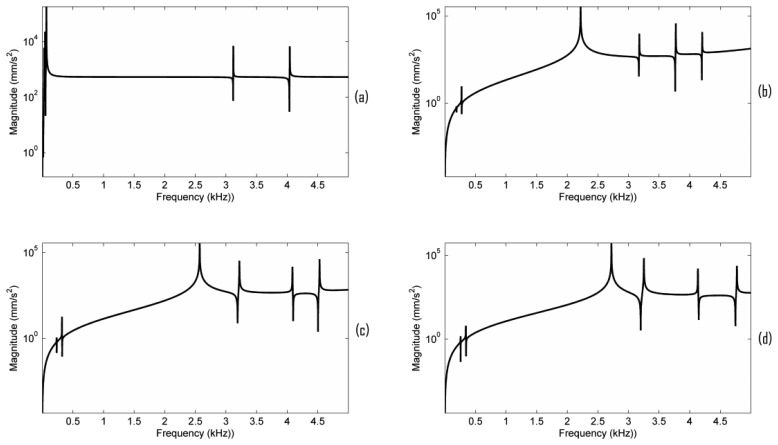
Frequency responses of the plate solid bridge with the damping ratio 0.017% critical when EFS1 = 0.01 N/mm^3^ and (**a**) EFS2 using the estimate ratios; (**b**) EFS2 = 1,000 N/mm^3^; (**c**) EFS2 = 1,600 N/mm^3^; (**d**) EFS2 = 2,000 N/mm^3^.

In Section 4.2, we have seen that the cut-outs in the bridge are important. In order to further examine this, the mobility analysis of a plate solid bridge as shown in Figure 1b was carried out under the same simulation configuration in Section 3. The plate solid bridge in Figure 1b has the same outline shape of the original bridge in Figure 1a. A few Y-directional acceleration responses measured at the bridge foot (using an average) in the sound-post side are presented in Figure 10, in which the damping ratio is set as 0.7% critical. These frequency responses are significantly different from the responses in Figure 5. Figure 11 shows the corresponding responses when the damping ratio is set as 0.017% critical. Our simulation results shows that no main resonance peak exists in the frequency range of 2–3 kHz if EFS1 > 0.01 N/mm^3^. Even if EFS1 ≤ 0.01 N/mm^3^, if we set EFS2 at the estimated ratios as used in Figures 5 and 6, no main resonance peak is observed as well in the frequency range of 2–3 kHz. A main resonance peak is only observed in the frequency range of 2–3 kHz when all the three of the following conditions are satisfied simultaneously through our trial-and-error: (1) EFS1 ≤ 0.01 N/mm^3^; (2) EFS2_G, EFS2_D, EFS2_A and EFS2_E are equal or very close; and (3) EFS2 in between 1,000 to 2,000 N/mm^3^. When EFS2 = 1,600 N/mm^3^ the peak is 2,569 Hz as shown in Figure 10c.

Notice that in Figure 10c, the ratio EFS2/EFS1 = 160,000 is much bigger than the estimated ratios in Figure 5. This condition seems difficult to achieve if fitting the plate bridge in a real violin because EFS1 and EFS2 are correlated each other as mentioned in Section 4.2. In addition, as shown in Figure 10b,d, this peak is very sensitive to the changing of EFS2. Thus, for the plate bridge, a main resonance peak in the frequency range of 2–3 kHz may exist but only EFS1 and EFS2 are under very strict conditions.

**Figure 12.**
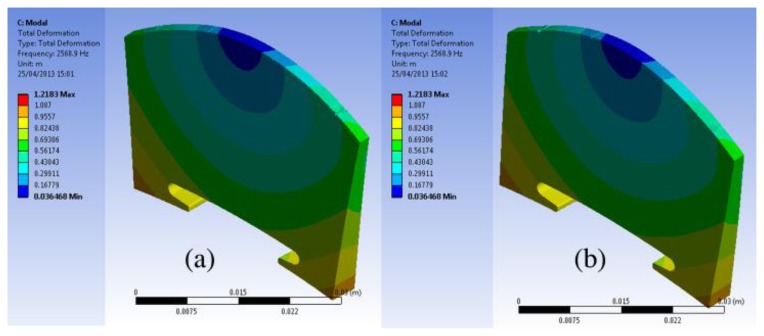
Bridge motion of the resonance mode at 2,569 Hz in Table 4. Note that we attach no significance to the amplitude of the motion.

In order to understand this finding, modal analysis was carried out for the plate solid bridge as shown in Table 4. Table 4 shows the modal analysis results when the contact stiffness EFS1 and EFS2 are set as in Figure 10. Comparing Tables 3 and 4, it can be seen that there are few resonance modes for the plate solid bridge. We then examined the motions of the resonance modes in Table 4. Figure 12 shows the motion of the resonance mode at 2,569 Hz in Table 4, which is an out-of-plane vibration mode. It can be seen that although the plate bridge could generate a main resonance peak in the frequency range of 2–3 kHz, the bridge motion is not the rocking motion.

## 5. Experimental Verification of EFS Impact on Bridge Mobility

In the literature, the mobility of an isolated bridge is normally measured with the bridge feet clamped in a vise [6,8]. However, in a real violin, the bridge is just put on top of the violin top plate with the string fitting on top of the bridge. Obviously, the bridge feet are not clamped when the bridge is fitted in a violin, so the mobility of a bridge measured with clamped feet is not accurate.

**Figure 13.**
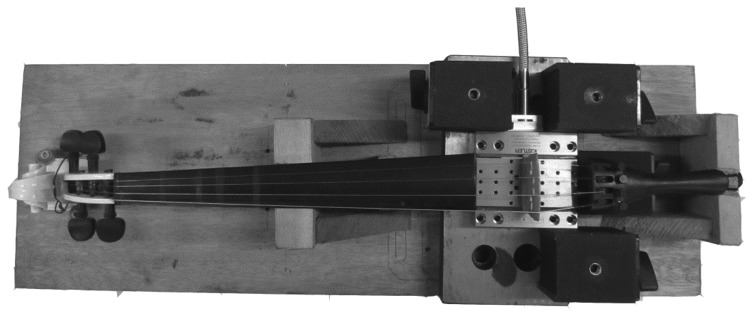
Experimental setup.

In order to verify the impact of the elastic foundation contact stiffness on bridge mobility, an experimental setup as shown in Figure 13 was built up. A KISTLER 9256C multi-component dynamometer was used to measure the 3D dynamic forces acted on the violin plate by the bridge feet. We disassembled a real violin and removed the body of the violin. The retained part of the violin was fixed on a wood board support, and the bridge was put on top of the dynamometer. The KISTLER 9256C dynamometer is designed for cutting force measurements in ultraprecise machining with a small design. It can measure the three orthogonal components of a force with high sensitivity (<0.002 N). The dynamometer has high natural frequency (f_n_(x) ≈ 5.1 kHz; f_n_(y) ≈ 5.5 kHz; f_n_(z) ≈ 5.6 kHz). Thus, this dynamometer is a good choice for our experimental measurement. Inside the dynamometer, four sensors are located underneath its top titanium plate in the four corners, and each sensors record the X-, Y-, and Z-components of the force. The dynamometer has 12 output channels. According to the experimental setup in Figure 13, we can obtain the three components of the force exerted to each bridge foot, the three components of the total forces exerted to the dynamometer by the bridge, and two moments of the force. Details refer to the user manual of the dynamometer. A sampling frequency of 20 kHz was used for data acquisition in the experiment.

In the experiment, the pitches of four steel strings were firstly tuned to the open string natural resonant frequencies, *i.e.*, G 196 Hz, D 294 Hz, A 440 Hz, and E 659 Hz. Then the dynamic forces exerted to the dynamometer by the bridge were collected when bowing a string. In order to obtain the frequency response of the bridge, we slide the finger in the fingerboard from the top nut down to the bridge continuously while bowing a string. Without finger slurring, the frequencies in the frequency spectrum of the recorded force consists of the natural resonant frequency and its harmonic series, *i.e.*, for the G-string 196 Hz, 392 Hz, 588 Hz,…, so that the frequency spectrum is discrete, as seen in Figure 5 of [16]. In such a discrete frequency spectrum, the resonances of the bridge mobility are difficult to observe. The finger slurring changes the fundamental resonance frequency continuously, and is equivalent to frequency sweeping in the frequency response analysis.

In the experimental setup of Figure 13, the violin bridge is equivalent to being mounted in a ‘rigid’ violin since the dynamometer can be treated as a rigid body. This setup facilitates the analysis of the dynamic forces exerted on the violin front plate by the bridge. In order to investigate the impact of dynamic contact stiffness on bridge mobility experimentally, we loosen or tighten the four strings simultaneously and then recorded the dynamic forces. Loosening the strings reduces the force F in Equation (1), thus reducing the EFS1 and EFS2. Tightening the strings increases EFS1 and EFS2. The force responses were further obtained by applying out the Fast Fourier Transform to these recorded dynamic forces.

**Figure 14.**
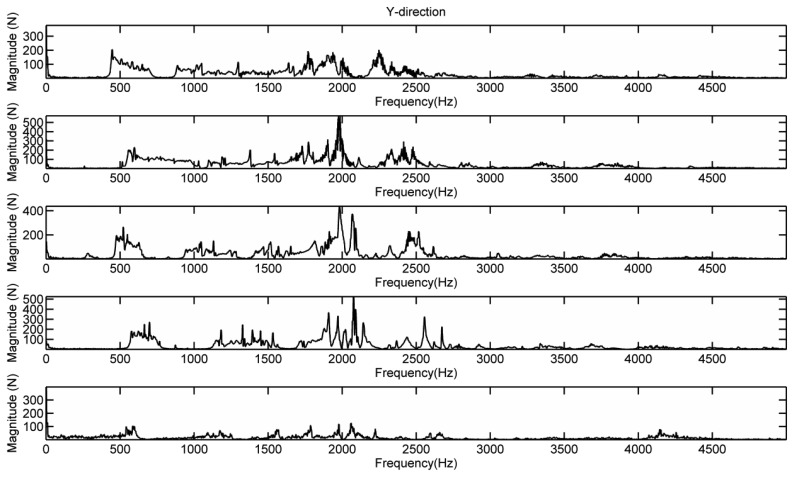
Y-directional force responses of the bridge. From upper to lower: lower 3, 2, and 1 tones, in tune, and higher 1 tone.

Figure 14 shows the Y-directional force responses of the bridge under different contact stiffness, respectively. The four strings were loosened simultaneously by one tone lower, two tones lower, and three tones lower. In order to not break the strings, we only tested the case of tightening the strings one tone higher. For each contact stiffness case, we record the dynamic forces when bowing the A-string with finger slurring. The force responses in Figure 14 were measured in the bridge foot close to the sound post. From Figure 14, it can be seen that, when loosening the strings the broad peak in the frequency range of 2–3 kHz becomes clearer than the string in tune. When tightening the string, the broad peak almost disappeared. Moreover, the broad-peak frequency is downshifted as EFS decreases. This experimental observation is in consistent with the simulation prediction in Section 4, confirming the impact of EFS on the bridge mobility.

## 6. Discussion and Conclusions

The bridge mounted in a violin has two contact interfaces: strings-bridge, and bridge feet-violin top plate. According to the Hertzian contact vibration theory, the changing contact stiffness in these interfaces can cause the bridge resonance frequency to shift and the resonance amplitude to change for each vibration mode. The mobility of an isolated bridge under in-plane excitation is first explored using the finite element method on the basis of the dynamic contact vibration model. Simulation results show that the contact stiffness has a significant impact on the bridge mobility. A novel experiment setup is then designed for the bridge mobility analysis. The experimental results confirmed the impact of the contact stiffness on the bridge mobility. The dynamic contact vibration model-based finite element method is demonstrated to be a powerful tool to study the bridge mobility.

It seems of interest to further explore in the future what is the impact of the dynamic contact stiffness on the overall dynamic response of a violin, particularly the bridge hill [17,18]. This can probably be done by modifying the finite element model to incorporate the violin corpus.

Moreover, the proposed experimental setup can be further modified, for example by removing the violin back plate and putting the dynamometer underneath the top plate, to study the effect of the contact vibration boundaries, such as the string tension, the roughness of the contact surfaces, and the area of the contact surfaces on the violin acoustical quality.

In addition, the Woodhouse model described in [4] provides a theoretical guidance for violin makers on how to trim the bridge in order to achieve the desired frequency, height and bandwidth of the bridge hill. It might be interesting as well to see if there is any link between the Woodhouse model and the dynamic contact vibration model.

## Figures and Tables

**Table 1.** Material properties of the violin bridge used in the simulation.

**Material**	**Density in Kg/m**^**3**^	**Young**'**s Modulus in MPa**			**Shear Modulus in MPa**			**Poisson**'**s Ratio**		

		**X**	**Y**	**Z**	**XY**	**YZ**	**XZ**	**XY**	**YZ**	**XZ**
Maple red	540	12,430	833	1,740	920	311	1,106	0.509	0.354	0.434

**Table 2.** The first ten-order natural resonant frequencies of the isolated bridge with the bridge feet clamped. O: out-of-plane vibration; I: in-plane vibration.

	**Frequency (Hz)**									

**Mode number**	**1**	**2**	**3**	**4**	**5**	**6**	**7**	**8**	**9**	**10**
Fixed constraint	515 (O)	1,080 (O)	1,441 (I)	1,629 (O)	2,771 (O)	4,166 (I)	4,384 (O)	5,195 (O)	5,697 (I)	6,822 (O)

**Table 3.** Modal analysis of the bridge in Figure 1a under the configuration of Figure 5. O: out-of-plane vibration mode; I: in-plane vibration mode.

**EFS1 (N/mm**^**3**^**)**	**Resonances in Hz**								
0.001	14 (I)	41 (I)	46 (I)	1,654 (O)	1,720 (O)	2,616 (I)	2,775 (O)	4,241 (O)	5,195 (O)
0.01	45	129	144	1,654	1,720	2,621	2,775	4,241	5,195
0.1	143	406	450	1,655	1,720	2,675	2,775	4,244	5,196
1	439	1,234	1,285	1,667	1,724	2,776	3,213	4,273	5,196
10	472	1,190	1,711	1,767	2,772	2,824	3,297	4,502	5,202
100	27	191	812	1,739	1,967	2,685	2,933	5,065	5,250
1,000	79	439	1,055	1,804	2,133	2,941	4,636	5,354	5,519
5,000	157	605	1,157	1,876	2,206	3,095	5,141	5,469	5,695

**Table 4.** Modal analysis of the plate solid bridge.

**EFS**	**Resonances in Hz**								
In Figure10a	0.02	2.7	3.6	30	45	66	3,134	4,047	6,759
In Figure10b	1.6	189	273	2,217	3,192	3,770	4,207	6,099	6,823
In Figure10c	2.0	233	321	2,569	3,233	4,089	4,522	6,850	7,178
In Figure10d	2.2	255	344	2,722	3,265	4,139	4,763	6,866	7,636
